# Relationship between risk perception and occupational accidents: a study among foundry workers

**DOI:** 10.1186/s42506-019-0025-6

**Published:** 2019-11-28

**Authors:** Mohammad Javad Jafari, Fahimeh Saghi, Ebrahim Alizadeh, Farid Zayeri

**Affiliations:** 1 Department of Occupational Health Engineering, School of Public Health and Safety, Shahid Beheshti University of Medical Sciences, Tehran, Iran; 2 Department of Applied Psychology, Faculty of Education and Psychology, Shahid Beheshti University, Tehran, Iran; 3 Proteomics Research Center and Department of Biostatistics, Faculty of Paramedical Sciences, Shahid Beheshti University of Medical Sciences, Tehran, Iran

**Keywords:** Accidents, Occupational, Perception, Risk

## Abstract

**Background:**

Risk perception is an effective factor in the determining of the incidence of unsafe behaviors and the occurrence of occupational accidents in the workplace.

**Objective:**

The purpose of this study is to determine the relationship between risk perception and occupational accidents among foundry workers in Tehran, Iran.

**Methods:**

In this cross-sectional study, all male workers in a foundry unit were initially selected (*n* = 245). After applying the study criteria and dismissing incomplete returned questionnaires, only 109 workers were included in the study. The General Health Questionnaire and the Flynn et al. Risk Perception Questionnaire were used to assess the mental health and risk perception of workers, respectively. Data regarding the occupational accidents of workers were also extracted from the accidents’ records of the foundry. Workers with a record of occupational accidents during 2013–2016 were compared with workers without an occupational accident record.

**Results:**

Findings showed that a one-unit increase in the risk perception score resulted into an increase of approximately 33% in the odds of the occurrence of occupational accidents, but this rate was not significant (*p* = 0.695). In addition, the study found no significant relation between the risk perception score and the frequency of occupational accidents (Spearman’s *r* = 0.003, *p* = 0.977).

**Conclusion:**

There is no statistically significant relationship between risk perception and occupational accidents among foundry workers in Tehran, Iran.

## Introduction

Occupational accidents are of major concern in both developed and developing countries because of their detrimental effects on health, society, and economy [1]. According to an estimation carried out by the International Labor Organization, approximately 340 million occupational accidents occur annually throughout the world [2]. Therefore, it is essential that preventive programs consider the causes of occupational accidents and the underlying factors affecting them.

Risk perception is defined as “an individual’s subjective judgment about the characteristics and severity of risks” [3]. Risk perception is one of the human features that affect an individual’s behavior in as such that the incorrect perception of risks is considered as a factor in the incidence of unsafe behaviors [4–6]. An increase in the level of an individual’s risk perception leads to an increase in the incidence of safe behaviors [4].

Studies have shown that unsafe behaviors are the major cause of occupational accidents [1, 7] and poor or incorrect perception of a workplace’s risks (or even the lack of risk perception) is a factor contributing to the incidence of occupational accidents [1, 3, 8].

During the casting process in a foundry, workers are exposed to various hazards (such as noise, pollution, vibration, or heat) or accidents (such as explosions, fires, or burns) [9]. The present study aimed at determining the relationship between risk perception and the occurrence and frequency of occupational accidents among workers of a foundry unit in Tehran, Iran, in 2016.

## Methods

### Study design and participants

In this cross-sectional analytical study, all male workers (*n* = 245) in a foundry unit in Tehran were initially included as the study population. The inclusion criteria were as follows: having a mental health score of 0–22 in the 28-item General Health Questionnaire, and having at least 3 years of job experience in the unit (as workers’ occupational accidents over 3 years will be investigated). The attrition effect of the workers was considered as study exclusion criteria. After applying the study criteria and dismissing incomplete returned questionnaires, only 109 workers were included in the study. Data was collected during June to September 2016.

### Tools

#### Questionnaires

The workers completed the questionnaires in the form of self-reporting. The 28-item General Health Questionnaire has been used to determine the severity of the individual’s mental disorders [10]. This questionnaire consists of four subscales: “somatic symptoms,” “anxiety,” “social dysfunction,” and “depression” [11]. The validity of the Persian version of this questionnaire (Nazifi et al.) showed that Cronbach’s alpha coefficient for the somatic symptoms, anxiety and insomnia, social dysfunction, severe depression, and questionnaire’s total scale were 0.865, 0.883, 0.746, 0.897, and 0.923, respectively [12]. In the study by Yaghoubi et al., [11] and Noorbala and Mohammad [13], the reliability coefficient of this questionnaire has also been reported as 0.88 and 0.91 respectively. The total score ranges from 0 to 84. A score of 0–22 in the 28-item General Health Questionnaire indicates that there is no (or minimum) level of mental disorder in the worker. A score range of 23–40 is considered mild, 41–60 is moderate, and 61–84 is considered as severe mental disorders [10].

The Flynn et al. Risk Perception Questionnaire has been used to assess an individual’s risk perception. This questionnaire consists of two major parts: (a) general information and (b) 133 questions divided into 10 separate sections (entitled in order as “job situation,” “the physical conditions of working environment,” “ the experience of risk-hazards,” “the probability of injury,” “the experience of risk-work tasks,” “job satisfaction,” “the assessment of safety,” “safety and accidents prevention,” “personal support and receiving help from others,” and “the experience of accidents and near-misses.” This questionnaire was validated by Soltani in Iran (2016). The content validity ratio, the content validity index, and Cronbach’s Alpha coefficient of this questionnaire were reported as 0.77, 0.96, and 0.93, respectively. The average of all scores for the domains of the Risk Perception Questionnaire was considered as the risk perception score. After evaluation, the worker receives a score of 1–5 (the minimum and maximum scores of the perception risk are 1 and 5, respectively) [14].

#### Records

The data related to the occupational accidents of workers (the occurrence of occupational accidents and the frequency of accidents) were extracted from the records of occupational accidents of the foundry. In order to obtain adequate information and generalizable inference, the data related to the occupational accidents over a consecutive 3-year period (2013–2016) was extracted. The workers with an occupational accidents’ record during 2013–2016 were compared with the workers without an occupational accident record (in terms of job category, age, and job experience).

#### Occupational accidents

In this study, occupational accident occurrence was defined as any occupational accident which has occurred (i.e., the occurrence or non-occurrence of accidents). The occupational accidents frequency for each worker was determined by counting the number of occupational accidents that had occurred over the past 3 years (from 2013 to 2016) for the individual.

### Statistical analysis

In this research, the studied variables were risk perception score, occupational accident occurrence, and occupational accident frequency. The collected data were analyzed using the IBM SPSS software, version 19.0. In order to describe the quantitative variables, the mean and standard deviation were used. The frequency distribution was also utilized to describe the categorical variables. The Kolmogorov-Smirnov test was used to assess the normality of the distribution of the variables. In order to compare the risk perception score between two groups of workers (injured and non-injured workers), the independent sample *t* test was used. Logistic regression was also applied to determine the relationship between risk perception score and the occurrence of occupational accidents. Regarding the normal distribution of risk perception score and the non-normal distribution of occupational accident frequency, the Spearman correlation coefficient was used to determine the association between risk perception score and occupational accident frequency. In all statistical analyses, *p* values less than 0.05 were considered as statistically significant.

## Results

### Participant characteristics

In this study, all workers (*N* = 109) were males. Most of them were married (93.6%) and the majority was educated up to the level of diploma (72.5%). The demographic variables of workers are presented in Tables 1 and 2.

**Table 1:** Descriptive statistics for quantitative demographic variables of foundry workers (*N* = 109) in Tehran, Iran, 2016

Variables	Min	Mean ± SD	Max
Age (year)	28	39.15 ± 5.22	57
Job experience^1^ (year)	3	12.84 ± 5.49	26
Work experience^2^ (year)	5	14.59 ± 5.07	26

^1^The duration of employment in the current job

^2^The duration of employment in the current job and other jobs

**Table 2:** Distribution of categorical demographic variables of foundry workers (*N* = 109) in Tehran, Iran, 2016

Variables	N (%)
Job type	
Machining	28 (25.7)
Sand cycle operator	4 (3.7)
Core production operator	3 (2.7)
Cast iron production operator	50 (45.9)
Shift supervisor	3 (2.7)
Melt operator	9 (8.3)
Technical and Maintenance	12 (11.0)
Education	
Below diploma	23 (21.1)
Diploma	79 (72.5)
Higher than diploma	7 (6.4)
Marital status	
Single	5 (4.6)
Married	102 (93.6)
Unknown^**1**^	2 (1.8)

^1^In the status of Unknown, workers have not specified their marital status in the questionnaire

### Occupational accidents

Table 3 shows the frequency distribution of workers by the occurrence of occupational accidents. Less than half of the workers (46.8%) had ever experienced occupational accidents.

**Table 3:** Distribution of foundry workers by occupational accident occurrence (*N* = 109) in Tehran, Iran, 2016

Occupational accident occurrence	*N* (%)
Yes	51 (46.8)
No	58 (53.2)
Total	109 (100.00)

Table 4 shows the frequency distribution of occupational accidents among injured workers in the foundry. The majority experienced one accident while none of the injured workers had experienced five or six occupational accidents.

**Table 4:** Distribution of the number of occupational accidents in the injured *foundry workers (*N *= 51) in Tehran, Iran, 2016*

Occupational accident frequency	*N* (%)
1	34 (66.67)
2	7 (13.72)
3	8 (15.69)
4	1 (1.96)
5	0 (0.00)
6	0 (0.00)
7	1 (1.96)

### Risk perception

The risk perception score is slightly higher among injured workers than those who had not been injured, but this difference was not statistically significant, as shown in Table 5.

**Table 5:** Descriptive statistics for foundry workers’ risk perception score by injury occurrence (*N* = 109) in Tehran, Iran, 2016

Injury	Min	Mean ± SD	Max	*p* value
Injured	2.32	2.84 ± 0.26	3.42	0.699
Not injured	2.29	2.82 ± 0.26	3.27	
Total	2.29	2.83 ± 0.26	3.42	

### Relationship between risk perception and occupational accidents

Table 6 shows the results of investigating the relationship between risk perception score and occupational accident occurrence. With a single unit increase in the risk perception score, the odds of occupational accident occurrence increased by about 33%; however, this rate was not statistically significant.

**Table 6:** Results of logistic regression analysis for assessing the relationship between risk perception score and occupational accident occurrence of foundry workers in Tehran, Iran, 2016

Independent variable	Coefficient β	Standard error	*p* value	Odds ratio	95% confidence interval
Risk perception score	0.29	0.742	0.695	1.337	0.312–5.724

In this study, the distribution of the workers’ risk perception score and the distribution of the workers’ occupational accident frequency was normal and non-normal, respectively. Thus, in order to determine the association between the risk perception score and occupational accident frequency, the Spearman correlation coefficient was used. This coefficient showed that there is no statistically significant relationship between the risk perception score and occupational accident frequency (Spearman’s *r* = 0.003, *p* = 0.977).

## Discussion

The present study was performed with the aim of determining the relationship between risk perception and occupational accidents among the workers of a foundry unit in Iran. The risk perception score of workers with an occupational accident record was higher than those without an occupational accident record. However, the observed difference was not statistically significant between both groups. One of the reasons for this non-significant difference may be the small sample size used in this study.

The results obtained by the present study are parallel to those obtained by Cezar-Vaz et al. who carried out a similar study among apprentice welders. Their results indicated that apprentice welders with an occupational accident record had higher risk perception than those without an occupational accident record [15]. In another study carried out by Cezar-Vaz et al., to identify risk perception and the types of occupational accidents among gas station workers, the perception of chemical risk was reported more among workers having records of chemical risk-related occupational accidents [16]. In a study done by Rundmo with the purpose of investigating the relationship between risk perception, job stress, and the frequency of accidents and near-accidents among petroleum personnel, it was observed that job stress and risk perception have a significant contribution in the rate of human errors and injuries (25% of the dependent variable variation was described by the independent variable (*R*^2^ = 0.25)) [17].

It is expected that by increasing an individuals’ risk perception, the occurrence of occupational accidents will be reduced [1]. Conversely, it should be taken into account that the occurrence of occupational accidents can also increase an individual’s risk perception [18].

### Study limitations

The relatively small sample size due to the screening of the workers in terms of mental health, the impossibility of evaluating the risk perception of workers before an occupational accident, and the inability to perform statistical tests to investigate the cause and time of accident occurrence due to the small sample size is considered as some limitations of the present study.

Risk perception is a human factor; hence, in order to investigate the relationship between risk perception and occupational accidents, it is proposed that only the accidents caused by human factors, i.e., unsafe acts, be considered. In addition, in order to obtain information about the effect of risk perception on occupational accident occurrence, it is suggested that the risk perception of individuals be assessed before the occurrence of occupational accidents.

## Conclusion and recommendation

The present study found no significant relationship between the risk perception score with the occurrence and frequency of occupational accidents. The researchers recommend conducting studies with a larger sample size to assess the aforementioned relationship with more accuracy.

Investigating the relationship between risk perception and occupational accidents and paying attention to this relationship in preventive programs could play an important role in the control of occupational accidents in the future.

## Data Availability

Data can be made available from the corresponding author upon request and with the permission of foundry authorities.
